# Effects of anodal transcranial direct current stimulation on postural stability in subacute stroke: A randomized control trial

**DOI:** 10.14814/phy2.70067

**Published:** 2024-09-30

**Authors:** Somia Shakeb, Mirza Obaid Baig, Turki Abualait, Sumaiyah Obaid, Woo‐Kyoung Yoo, Shahid Bashir

**Affiliations:** ^1^ Faculty of Rehabilitation & Allied Health Sciences Riphah International University Islamabad Pakistan; ^2^ College of Applied Medical Sciences Imam Abdulrahman Bin Faisal University Dammam Saudi Arabia; ^3^ Department of Physical Medicine and Rehabilitation, Hallym University Sacred Heart Hospital Hallym University College of Medicine Anyang Korea; ^4^ Neuroscience Center King Fahad Specialist Hospital Dammam Saudi Arabia

**Keywords:** anodal transcranial direct current stimulations, neuromodulation, neuroplasticity, postural control, primary motor cortex, stroke

## Abstract

Anodal transcranial direct current stimulation (tDCS) promotes neuromodulation and neuroplasticity in the brain. The aim of this study was to determine the long‐term effects of the anodal tDCS on postural and trunk stability, physical performance, anticipatory postural adjustment and quality of life in sub‐acute stroke patients. Thirty‐six participants with sub‐acute stroke were divided into experimental and control groups using sealed envelope randomization. Outcome measures comprised the Postural Assessment Scale for Stroke, Trunk Impairment Scale, Time Up and Go Test, Functional Reach Test, and Stroke‐Specific Quality of Life Scale. Assessments were conducted at 0, 3, 6, 9, and 12 weeks. Within‐group analysis revealed significant improvement in both the experimental (*p*‐value < 0.05) and control groups (*p*‐value < 0.005). Notably, significant effects were observed in postural stability after intervention, and during one of the detraining assessments, the experimental group showed superior results compared to the control group in subacute stroke. Anodal tDCS yield significant short‐ and long‐term effects on postural stability, while short term effects on trunk stability. Additionally, long term effects were observed on the physical performance and anticipatory postural adjustments while no effects at quality of life either short or long term basis among the subacute stroke patients.

## 1 INTRODUCTION

Stroke is one of the primary causes of mortality and disability (Stinear et al., 2020) and the second leading cause of death and disability globally in 2019 (6.6 million fatalities and 143 million disability‐adjusted life years lost). Stroke incidence, prevalence, mortality, and disability‐adjusted life years lost worldwide increased by 70%, 85%, 43%, and 32% over the past 30 years, respectively (Owolabi et al., 2022).

Stroke patients develop different symptoms, which depend on the location of cerebral injury, including motor, sensory, perceptual, and cognitive impairments. Motor impairments, constitute of 80% in stroke survivors, greatly affect their activities and participations (Estrada‐Barranco et al., 2021). Postural instability is frequently present in 83% of patients with acute stroke, with an increase in fall tendency by 73% (Hugues et al., 2017). Patients experience limitations that influence their ability to do activities of daily living, which affects their functionality and quality of life (Langhorne et al., 2009).

It is necessary to incorporate therapy modalities aimed at enhancing postural and trunk control, and weight‐bearing symmetry. Following stroke, cortical map reorganization is crucial for much of the gross functional recovery and noninvasive brain stimulation (NIBS) techniques can induce cortical neuroplastic changes by altering the neuronal activity and modulating cortical excitability, which have potential behavioral effects and might encompass therapeutically benefits for neurorehabilitation following cortical lesion (Horn & Fox, 2020).

Transcranial direct current stimulation (tDCS) is one of the noninvasive brain stimulation techniques that is painless, simple, and safe to be administered in patients with stroke. The motor cortex's excitability and activity are polarity‐specifically modulated by tDCS, the anode or cathode depolarization or hyperpolarization of resting membrane potentials causes the regulation of excitability (Farnad et al., 2021). Evidence has shown the therapeutic benefits of applying multiple sessions of tDCS following stroke. A six‐week strategy combining conventional care and 20‐min anodal tDCS treatments can enhance gait speed, trunk mobility, upper extremity function, and quality of life in subacute stroke patients (Jamil & Nitsche, 2017). A meta‐analysis showed that stroke patients with limb impairment could recover effectively with tDCS (Taud et al., 2021). Bi‐hemispheric tDCS is a viable strategy when used in conjunction with task‐oriented training to speed motor recovery in strokes (Agboada et al., 2020).

Although numerous studies postulate the immediate and short‐term effects of the anodal tDCS on postural stability in subacute stroke, there is very little literature available regarding the long‐term effects of tDCS on motion perception, motor skill learning, etc. but not at the postural stability, up to the author's limited access, therefore this study aimed to determine the long‐term as well as short‐term effects of tDCS on postural and trunk stability, physical functions, anticipatory postural adjustments and quality of life in subacute stroke patients. We hypothesize that the tDCS may affect the postural, trunk stability that leads to the improvement in the physical functions, postural adjustments, and quality of life as well among the stroke patients because the subacute phase of stroke has the chances of motor recovery while inducing the neural connectivity and plasticity.

## 2 METHODS

### 2.1 Ethics committee approval statement

The institutional research ethics committee of Riphah International University with ref. no (RIPHAH/RCHS/REC/LETTER‐01361) before the experiment was started and that has been conducted in accordance with the principles set forth in the Helsinki Declaration has approved the study.

### 2.2 Study setting & subjects

#### 2.2.1 Clinical trials

A randomized control trial (RCT) was conducted from December 2022 to June 2023 with the ethical approval and registered at clinicaltrial.gov with the unique ID: NCT 05903599. After being written informed consent, all participants were randomly allocated into experimental and control groups through the sealed envelope method. The envelopes were signed, sequentially numbered, and remained sealed until the patient gave their consent to participate in the study. The envelope was then opened, and the patient was allocated accordingly to either the experimental or control group.

The participants were recruited through the nonprobability convenient sampling method having the following characteristics: both sexes aged between 45 and 65 years, sub‐acute ischemic stroke with a minimum score of 18 on Postural Assessment Scale for Stroke (PASS). The exclusion criteria were hearing and visual loss/deficit, recurrent stroke, any medical condition affecting the trunk/postural stability, wound at a skull, presence of shunt/or metallic implant at the cranial region, brain tumor, musculoskeletal conditions/surgery in the lower extremities, cognitively compromised, and patients with pacemaker.

The sample size was determined using G‐power 3.1.9.4 software through a priori model application. The calculated sample size was 36, considering the power of 0.95, with the effect size of 0.25, and alpha of 0.05, divided into two groups each group contained 18 participants.

#### 2.2.2 Intervention

##### Experimental group

A tDCS device (Soterix Medical 1 × 1 Transcranial Electrical Stimulation) with an electrode size of 5 × 5 cm covered by saline‐soaked sponges were used to deliver the 2 mA anodal tDCS for 20 min to the primary motor cortex. The ramp‐up and down period was set at 30 s to minimize the side effects. The anode was placed at the primary motor cortex on the ipsilesional side and the cathode on the supraorbital region contralesionally.

The head‐specific measurements of participant's heads following the 10/20 EEG guidelines and training previously provided (Klem, 1999). The C3 and C4 areas were localized and the distances between left preauricular notch to C3 and right preauricular notch to C4 was measured consistent with clinical practices for localizing C3 and C4 (Teplan, 2002).

Conventional treatment of postural stability consists of postural training by visual feedback, trunk control exercises, and a task‐oriented approach (Bornheim et al., 2022). The experimental group received the active anodal tDCS with the conventional treatment while the control group had the sham stimulation with the conventional treatment as mentioned.

##### Control group

The duration of the sham stimulation, by using the same Soterix Medical 1×1 Transcranial Electrical Stimulation, was 20 min opting the sham option of the device. This maneuver gives placebo effects on the patient (Verheyden et al., 2004). Conventional treatment of postural stability consists of postural training by visual feedback, trunk control exercises, and a task‐oriented approach (Bornheim et al., 2022). The control group had the sham stimulation with the conventional treatment as mentioned.

The participants received the intervention, of 60 min (20 min stimulation and 40 min of conventional treatment), 5 days a week for 6 weeks, and the assessment was made at baseline, week 3rd, 6th, 9th, and 12th. Every participant was briefed well about the study protocols, possible benefits, and adverse effects as well. All the participants have given written informed consent prior the participation in the study.

#### 2.2.3 Outcome measures

The PASS as primary outcome and the secondary outcome included the Trunk Impairment Scale (TIS), functional reach test (FRT), timed up and go (TUG) test, and stroke‐specific quality of life (SS‐QoL) were used as outcome measures for postural stability, truncal stability, anticipatory postural adjustments, physical function, and quality of life respectively at baseline, week 3, 6, 9, and 12.

##### Postural Assessment Scale for Stroke (PASS)

It measures the functional abilities and postural imbalance of stroke patients in a variety of situations, including lying, sitting, standing, and changing positions. There are 12 items on this scale with a total score of 36, with 0 being the least functional and 3 the most functional status (Merchán‐Baeza et al., 2014). The PASS contains a high level of accuracy in stroke patients with reliability and correlation coefficient between measures of global score are 0.99 and 0.98, respectively (Mazzoleni et al., 2019).

##### Trunk Impairment Scale (TIS)

TIS assesses the motor impairment of the trunk, following a stroke, including the static and dynamic seated trunk stability as well as trunk coordination on a scale from 0 to 23 at seven items (Liang et al., 2020). TIS had a good and comparable responsiveness of 0.94 (Ahmad et al., 2022).

##### Functional reach test (FRT)

FRT is a method for clinical outcome measurement and assessment that can quickly determine anticipatory postural control. The patient is told to stand close to a wall, but not touch it, and hold a closed fist with the shoulder closest to the wall flexed 90 degrees. On the yardstick, the assessor marks the beginning position at the third metacarpal head. Give the patient instructions to reach as far forward as possible without taking a step. It is noted where the third metacarpal is located. Scores are calculated by comparing the start and end positions in the reach distance, which is often expressed in inches. There should be three trials, with the average of the final two being reported. FRT has an interrater agreement of 0.98 for each measurement and a test–retest reliability of 0.89 (Shah et al., 2021).

##### Timed up and go test (TUG)

The TUG test is a general physical performance test used to evaluate locomotion, mobility, and balance in people with balance disorders. The patients are instructed to get out of their chairs and walk at a normal pace to a queue that is three meters long before turning around and walking back to a chair where they can sit down, and the time would be recorded for the performance from standing to sit again on the chair. The TUG test has an intraclass correlation coefficient of 0.98, making it a trustworthy test for evaluating balance in stroke patients (Tien et al., 2020).

##### Stroke specific quality of life (SS‐QoL)

SS‐QoL is a valid and self‐reported health‐related quality of life tool specifically designed for stroke survivors. It has 49 items over 12 categories, including 5 items each on mobility, energy, and upper extremity function. Higher scores indicate better functioning in the domains of work/productivity, mood, social roles, family roles, language, thinking, and personality (Wang et al., 2022). The test–retest results demonstrated remarkable stability, with Spearman's *r* = 0.65–0.99 and cronbach's alpha, for internal consistency, between 0.8 and 0.94 for all domains (Klomjai et al., 2022).

### 2.3 Statistical analysis

All the data were analyzed using IBM SPSS version 25 for the per protocol (PP) analysis. The data was not normally distributed, analyzed by Shapiro Wilk test, for PASS, TUG, and FRT while TIS and SS‐QoL were normally distributed. Friedman and Mann–Whitney tests were used as nonparametric categories for between and within the group respectively. The independent *t*‐test and repeated measure ANOVA were used for normally distributed variables. The baseline characteristics of both groups were analyzed by independent sample *t*‐test for age and chi‐square for the rest of the demographics.

## 3 RESULTS

A total of 36 participants were randomly allocated into the experimental (*n* = 18) and control (*n* = 18) with the mean age of 54.38 and 58 years, respectively. The sample contains more males than females and stroke duration of 4.88 and 4.97 months in the experimental and control group respectively. The baseline characteristics of both groups are present in Table 1.

**TABLE 1:** Baseline characteristics of both groups.

Variables	Experimental group	Control group	*p*‐Value
Age (years)	54.38 ± 7.45	58.00 ± 9.16	0.205
Gender; *n* (%)			
Male	13 (72.2)	10 (55.6)	0.232
Female	5 (27.8)	8 (44.4)	
Hemiplegic site			
Right	10 (55)	12 (66.7)	0.640
Left	8 (45)	6 (33.3)	
Stroke type			
Ischemic	18 (100)	18 (100)	0.234
Hemorrhagic	0 (0)	0 (0)	
Stroke duration in months (mean ± SD)	4.88 ± 1.01	4.97 ± 0.81	0.675
Comorbidities; *n* (%)			
HTN	3 (16.6)	7 (38.8)	0.312
HTN + DM	14 (77.7)	10 (55.5)	
None	1 (5.7)	1 (5.7)	

Abbreviations: DM, Diabetes Mellitus; HTN, Hypertension; SD, Standard deviation.

There was no significant difference at the baseline for all the variables, between the groups before the intervention (*p* > 0.05). After the intervention, there were significant effects in PASS at week 6th and 9th while there were no significant effects in TUG, SS‐QoL at all time points (*p* > 0.05). For the FRT outcome measure, a statistically significant was found at week 12th. The intervention showed significant effects for TIS at week 3rd and 6th with *p* = 0.034 and 0.029, respectively (Tables 2 and 3). Additionally, almost all the determinants showed no statistically significant effects in the detraining phase (week 9th to 12th) except the PASS at week 9th (*p* < 0.001) and FRT at week 12 (*p* = 0.010). Both groups showed significant effects when compared to the baseline (*p* < 0.001) (Tables 4 and 5). The further analysis yielded the effects of interventions in both groups were significant (*p* < 0.001) at all‐time points except the detraining phase. The effect size measurement yielded the Cohen's d of 2.09 and eta squared of 0.52, indicating the large effect size (Cohen, 2013).

**TABLE 2:** Between group analysis for PASS, TUG & FRT.

Variable	Time	Group	Mean rank	*p*‐Value
PASS	0 week	Experimental	16.53	0.265
		Control	20.47	
	3rd week	Experimental	21.14	0.134
		Control	15.86	
	6th week	Experimental	23.89	**0.002**
		Control	13.11	
	9th week	Experimental	24.78	**<0.001**
		Control	12.22	
	12th week	Experimental	18.56	0.988
		Control	18.44	
TUG	0 week	Experimental	18.0	0.791
		Control	19.0	
	3rd week	Experimental	16.86	0.355
		Control	20.14	
	6th week	Experimental	15.28	0.068
		Control	21.72	
	9th week	Experimental	13.94	0.10
		Control	23.06	
	12th week	Experimental	15.22	0.064
		Control	21.78	
FRT	0 week	Experimental	18.83	0.849
		Control	18.17	
	3rd week	Experimental	19.36	0.628
		Control	17.64	
	6th week	Experimental	20.50	0.265
		Control	16.50	
	9th week	Experimental	21.86	**0.055**
		Control	15.14	
	12th week	Experimental	23.00	**0.010**
		Control	14.00	

*Note:* Bold values indicate significance of *p* < 0.05.

Abbreviations: FRT, Functional reach test; PASS, Postural Assessment Scale for Stroke; TUG, Timed up and go test.

**TABLE 3:** Between group analysis for TIS & SS‐QOL.

Variable	Time	Group	Mean ± SD	*p*‐Value
TIS	0 week	Experimental	10.78 ± 2.2	0.362
		Control	11.72 ± 3.6	
	3rd week	Experimental	13.67 ± 2.2	**0.034**
		Control	12.67 ± 2.8	
	6th week	Experimental	16.89 ± 2.3	**0.029**
		Control	14.11 ± 3.0	
	9th week	Experimental	17.72 ± 2.5	0.241
		Control	15.05 ± 2.6	
	12th week	Experimental	17.22 ± 2.5	0.455
		Control	16.94 ± 3.0	
SS‐QoL	0 week	Experimental	134.24 ± 25.20	0.193
		Control	136.38 ± 31.74	
	3rd week	Experimental	160.60 ± 26.41	0.205
		Control	146.83 ± 30.90	
	6th week	Experimental	184.50 ± 31.60	0.345
		Control	156.77 ± 33.44	
	9th week	Experimental	205.55 ± 38.28	0.961
		Control	170.55 ± 33.52	
	12th week	Experimental	215.66 ± 37.99	0.794
		Control	175.27 ± 34.82	

*Note:* Bold values indicate significance of *p* < 0.05.

**TABLE 4:** Within group analysis for PASS, TUG & FRT.

Variable	Time	Experimental group			Control group		
		Mean rank	Median (IQR)	*p*‐Value	Mean rank	Median (IQR)	*p*‐Value
PASS	0 week	1.06	21 (9)	**<0.001**	1.0	22.0 (3.0)	**<0.001**
	3rd week	2.12	28 (5)		2.0	25.5 (7.75)	
	6th week	3.09	32 (2.5)		3.0	28.5 (7.75)	
	9th week	3.74	36 (0.5)		3.9	30.5 (6.75)	
	12th week	4.00	34 (6.25)		4.02	35 (7)	
TUG	0 week	4.83	31 (70)	**<0.001**	5.00	30 (68.8)	**<0.001**
	3rd week	3.83	24 (52)		3.83	25 (64)	
	6th week	2.78	17 (26)		2.94	23 (56)	
	9th week	1.72	11 (14)		2.06	22 (45)	
	12th week	1.83	16 (44.5)		1.17	21 (45)	
FRT	0 week	1.14	5.4 (3.6)	**<0.001**	1.22	3.75 (7.5)	**<0.001**
	3rd week	2.03	6.7 (4)		2.11	4.4 (7.6)	
	6th week	3.06	9 (3.6)		3.00	4.75 (7.7)	
	9th week	3.94	11.4 (4.5)		3.97	6.3 (7.73)	
	12th week	4.83	12.8 (3.55)		4.69	6.7 (7.9)	

*Note:* Bold values indicate significance of *p* < 0.05.

Abbreviations: FRT, Functional reach test; PASS, Postural Assessment Scale for Stroke; TUG, Timed up and go test.

**TABLE 5:** Within group analysis of TIS & SS‐QoL.

	Time	Experimental group			Control group		
		Mean ± SD	Measure of effect	*p*‐Value	Mean ± SD	Measure of effect	*p*‐Value
TIS	0 week	10.23 ± 3.07	0.887	**<0.001**	10.56 ± 3.8	0.845	**<0.001**
	3rd week	13.82 ± 2.15			12.31 ± 2.9		
	6th week	17.23 ± 1.88			14.5 ± 2.9		
	9th week	18.11 ± 1.99			15.5 ± 2.2		
	12th week	17.59 ± 2.06			17.69 ± 2.24		
SS‐QoL	0 week	136.29 ± 24.4	0.914	**<0.001**	140.93 ± 30.7	0.855	**<0.001**
	3rd week	163.52 ± 22.4			152.68 ± 27.4		
	6th week	189.47 ± 20.3			163.87 ± 27.9		
	9th week	211.76 ± 28.6			179.37 ± 22.9		
	12th week	222.4 ± 22.9			184.68 ± 22.9		

*Note:* Bold values indicate significance of *p* < 0.05.

Abbreviations: SS‐QoL, Stroke specific quality of life; TIS, Trunk Impairment Scale.

## 4 DISCUSSION

The main aim of this study was to ascertain the short‐ and long‐term benefits of anodal tDCS on patients with subacute stroke in terms of postural stability, trunk stability, physical functions, anticipatory postural adjustment, and quality of life in patients of sub‐acute stroke by comparing the experimental and control groups. The experimental group received anodal tDCS with conventional treatment and the control group received sham stimulation with conventional treatment. It was observed that there were no statistically significant differences present between the experimental group and the control group. Only the postural stability on PASS showed significant results after intervention as well as one follow‐up result was significant between the groups. Within‐group analysis which was done by using the Friedman test showed significant improvement on PASS, TUG, and FRT. Similarly, within‐group analysis which was done by using the RM‐ANOVA test showed significant effects on TIS and SS‐QoL.

The evidence of anodal tDCS on motor cortical area or M1 region is effective for functional stroke rehabilitation, taken from 46 different double‐blinded, sham‐controlled, and randomized trials with overall good quality, but because of the difference of stimulation parameters, outcome tools, and the demographics of the patients affects the results (Bornheim et al., 2022). S‐Mazzolini and VD Tran investigated the effects of anodal tDCS on motor recovery in subacute stroke and found no significant differences between the two groups, tDCS and wrist robotic therapy. There was a significant increase in movement velocity and smoothness, but no significant difference was observed between groups. The combination of both treatments did not demonstrate additional effects compared to wrist robot‐assisted training alone in subacute stroke patients (Mazzoleni et al., 2019). tDCS has shown efficacy in the recovery of stroke patients with limb dysfunction following hemispheric cerebral vascular accidents (CVA), necessitating distinct parameters for upper and lower limb rehabilitation. Anodal tDCS has been beneficial in enhancing upper limb function recovery in chronic stroke patients with treatment sessions numbering ten or fewer, while subacute patients have exhibited improved lower limb functions (Cohen, 2013). However, studies evaluating the effects of anodal tDCS on postural stability in stroke patients using computerized dynamic posturography found no significant impact of stimulation (Liang et al., 2020). There are contrasting results regarding the effects of anodal tDCS on balance, physical performance, gait, and the risk of falls among stroke survivors (Ahmad et al., 2022). Additionally, its effects on postural stability remain inconclusive (Wang et al., 2022). Klomjai et al., 2022, reported no significant effects of anodal/cathodal tDCS between the groups but within‐group differences were found, dual treatment (anodal and cathodal tDCS) affects greater than any other treatment (Klomjai et al., 2022). Anodal tDCS improved the strength of the lower limb muscles lead to control the lower limb movement (Bai et al., 2019), leg region of the afflicted primary motor cortex observed a substantial increase in knee extensor peak torque following anodal tDCS, as compared to the baseline measurement. (Marangolo et al., 2014). Shah et al., 2021 reported the effects of tDCS on balance and SS‐QoL in stroke patients that the balance improved after the treatment with cathodal, anodal and sham stimulation but SS‐QoL showed no significant effects in any of the three types of stimulation (Shah et al., 2021). Quality of life was seen improving in patients receiving anodal tDCS of stroke who received 3 months of tDCS (Verheyden et al., 2004). The treatment with anodal tDCS did not improve motor function and upon the subgroup analysis showed a significant effect of tDCS on motor functions in the acute and subacute phases with impact at SS‐QoL was very low (Sánchez‐León et al., 2021).

As this study concluded that anodal tDCS has no significant short‐ and long‐term effect on anticipatory postural control, Bruttini et al. reported no improvement in anticipatory postural control (Lima et al., 2023) Frank et al. suggested, in contrast, that those patients who received anodal tDCS had significant improvement in their anticipatory control in stroke patients (Khallaf, 2020) while Nambu et al. stated that postural control was seen more improved at later stages of rehabilitation compared in the early phase of recovery (Jamil & Nitsche, 2017).

The evidence shows that the intervention based on conventional treatment and 20 mins of anodal tDCS sessions for six weeks can improve trunk mobility, upper extremity function, gait speed and quality of life in subacute stroke, trunk mobility and quality of life, both showed significant improvement in within group analysis (Benaim et al., 1999). Another meta‐analysis suggests that tDCS is effective for the recovery of stroke patients with limb dysfunction. The parameters of tDCS are different for upper limb and lower limb dysfunction. (Fujiwara et al., 2004). There is a study which suggests that bihemispheric tDCS is a promising approach in combination with task‐oriented training for facilitating motor recovery in subacute stroke patients (Williams et al., 1999). The initial studies, like the current study, used anodal tDCS before motor treatment and shown that there was a considerable functional improvement of the paretic side when compared to therapy alone (Liang et al., 2020). Physical performance was improved by either peripheral stimulation or tDCS alone, but the combination was superior to both. In a separate study, ten subacute stroke patients received concurrent occupational and physical therapy for two 5‐day bi‐hemispheric tDCS intervention periods. Bihemispheric tDCS and peripheral sensorimotor stimulation were observed to improve upper‐extremity Fugl Meyer ratings. Additionally, they showed that the results of numerous treatment sessions in individuals with chronic stroke may not always have a linear response function. (Lima et al., 2023). A study investigated the effects of tDCS and functional electrical stimulation in trunk acceleration during gait training in stroke patients. The effects of anodal tDCS were significant, the effects of functional electrical stimulation were nonsignificant, and the effects of both treatments were also significant (Mitsutake et al., 2021).

With regard to the long‐term effects observed, anodal stimulation produced significant after‐effects in PASS at week 6th and 9th, and improvement in the FRT outcome measure at week 12th suggesting that tDCS has an after‐effect. Such effects might be because tDCS modulates cortical excitability and induce neural plasticity mechanisms (Knotkova et al., 2019; Nitsche et al., 2003) via effects on synaptic long‐term potentiation (LTP) and long‐term depression (LTD) (Nitsche et al., 2012) which prolong the stimulation after‐effects. tDCS persuades complex changes in the brain includes perturbation in functional connectivity (Polanía et al., 2012), synchronization within multiple brain regions (Mares et al., 2022; Polanía et al., 2012) and alterations in the neurotransmitter systems (Nitsche et al., 2009; Yamada & Sumiyoshi, 2021). Moreover, tDCS induces tissue biochemical changes by the flow of the electric current which contributes to the process of neural plasticity that help in boosting functional recovery (Yamada & Sumiyoshi, 2021). Although the results of this study are not very encouraging, between‐group analysis showed nonsignificant results, but within‐group and post hoc analysis showed significant improvement. Besides that, several concerns and limitations should be addressed in follow‐up research. The findings do, however, contribute to the growing body of research suggesting that brain stimulation may be an effective adjunct treatment for stroke rehabilitation.

### 4.1 Limitations

Few limitations are directly related to the effects of anodal tDCS for postural stability in subacute stroke because the three stages of stroke may affect the influence of interventions and should be considered. The second shortcoming was the inability of neuroimaging brain activity, responses during and after the stimulation. Lastly, the usage of subjective tools for postural assessment instead of objective measurement as the author did not have the access, funding to avail the posturography, force plate, etc. for the postural assessment.

## 5 CONCLUSION

The results indicate that patients with subacute stroke who received anodal tDCS did not exhibit intervention effects during the intervention phase for postural stability, physical performance, anticipatory postural adjustments, and quality of life. However, promising effects were observed for truncal stability. Furthermore, during the detraining/long‐term phase, there were no intervention effects across all variables, but both groups showed significant effects within their respective groups. It is recommended to study the effects of anodal tDCS by using objective tools such as functional MRI, EEG, thus the neuroplastic effects can be elucidated. It is recommended to study all three stages of stroke together so the results can be compared.

## AUTHOR CONTRIBUTIONS

All authors read and approved the final version of the manuscript. All authors (SS, MOB, TA, SO, WKY, and SB) contributed equally to this work. They were all involved in drafting and revising the manuscript critically for important intellectual content.

## FUNDING INFORMATION

No funding available for this work.

## CONFLICT OF INTEREST STATEMENT

Neither of the authors has any conflict of interest to disclose.

## ETHICS STATEMENT

The study was carried out in accordance with the code of international and local Ethics (Declaration of Helsinki). This study was with the ethical approval of Research Ethical Committee of Riphah International University (RIPHAH/RCHS/REC/LETTER‐01361) and registered at clinicaltrial.gov with the unique ID: NCT 05903599.

## Data Availability

The datasets used and/or analyzed during the current study are available from the corresponding author on reasonable request.
